# Effect of vitamin D monotherapy on indices of sarcopenia in community‐dwelling older adults: a systematic review and meta‐analysis

**DOI:** 10.1002/jcsm.12976

**Published:** 2022-03-08

**Authors:** Konstantinos Prokopidis, Panagiotis Giannos, Konstantinos Katsikas Triantafyllidis, Konstantinos S. Kechagias, Jakub Mesinovic, Oliver C. Witard, David Scott

**Affiliations:** ^1^ Department of Musculoskeletal Biology, Institute of Life Course and Medical Sciences University of Liverpool Liverpool UK; ^2^ Society of Meta‐research and Biomedical Innovation London UK; ^3^ Department of Life Sciences, Faculty of Natural Sciences Imperial College London London UK; ^4^ Department of Nutrition & Dietetics Musgrove Park Hospital, Taunton & Somerset NHS Foundation Trust Taunton UK; ^5^ Department of Metabolism, Digestion and Reproduction, Faculty of Medicine Imperial College London London UK; ^6^ Institute for Physical Activity and Nutrition (IPAN), School of Exercise and Nutrition Sciences Deakin University Burwood Victoria Australia; ^7^ Department of Medicine, School of Clinical Sciences at Monash Health Monash University Clayton Victoria Australia; ^8^ Centre for Human and Applied Physiological Sciences, Faculty of Life Sciences and Medicine King's College London London UK

**Keywords:** Vitamin D, Frailty, Sarcopenia, Handgrip strength, Physical performance, Older adults

## Abstract

**Background:**

Vitamin D supplementation is proposed as a potentially effective nutritional intervention to mitigate the risk of sarcopenia. The aim of this systematic review and meta‐analysis was to investigate the impact of vitamin D supplementation monotherapy on indices of sarcopenia in community‐dwelling older adults.

**Methods:**

A comprehensive search of the literature was conducted in PubMed, Web of Science, Scopus, and Cochrane Library. Eligible randomized controlled trials (RCTs) compared the effect of vitamin D supplementation (as monotherapy) with placebo on indices of sarcopenia in older (>50 years) adults. Using the random effects inverse‐variance model, we calculated the mean difference (MD) in handgrip strength (HGS), short physical performance battery (SPPB), timed up and go (TUG), and appendicular lean mass (ALM) between groups. We also calculated the standardized mean difference (SMD) in general muscle strength and general physical performance (composite plot of all muscle strength and physical performance outcomes, respectively) between groups.

**Results:**

Ten RCTs were included in the meta‐analysis. A significant decrease in SPPB scores was observed with vitamin D supplementation compared with placebo (MD: −0.23; 95% CI −0.40 to −0.06; *I* ^*2*^ = 0%; *P* = 0.007). Vitamin D supplementation conferred no effect on HGS (MD: −0.07 kg; 95% CI −0.70 to 0.55; *I* ^*2*^ = 51%, *P* = 0.82), TUG (MD: 0.07 s; 95% CI −0.08 to 0.22; *I* ^*2*^ = 0%, *P* = 0.35), ALM (MD: 0.06 kg/m^2^; 95% CI: −0.32 to 0.44; *I* ^*2*^ = 73%, *P* = 0.77), general muscle strength (SMD: −0.01; 95% CI −0.17 to 0.15; *I* ^*2*^ = 42%, *P* = 0.90), or general physical performance (SMD: −0.02; 95% CI −0.23 to 0.18; *I* ^*2*^ = 71%, *P* = 0.83).

**Conclusions:**

Vitamin D supplementation did not improve any sarcopenia indices in community‐dwelling older adults and may compromise some aspects of physical performance. Future studies are warranted to investigate the impact of vitamin D supplementation on individual indices of SPPB, including mobility and balance, in older adults.

## Introduction

Optimal body composition and skeletal muscle function are key contributors to healthy ageing. Ageing is associated with the gradual loss of muscle mass and strength called sarcopenia,^1^ which leads to increased risk of falls and fractures, hospitalization, immobilization, and mortality rates.^2^ Numerous non‐pharmacological interventions including resistance exercise and protein, creatine, n‐3 polyunsaturated fatty acid, and vitamin D supplementation have been studied with the aim to reduce the prevalence of sarcopenia.^3^, ^4^, ^5^ Preliminary evidence suggests that vitamin D supplementation improves muscle mass and strength in older adults; however, most studies that have investigated the impact of vitamin D supplementation on musculoskeletal outcomes have been conducted in combination with structured exercise, whey protein, and/or calcium supplementation with no consideration of co‐morbidity status.^6^, ^7^ Hence, the effect of vitamin D supplementation as a monotherapy on musculoskeletal health outcomes in community‐dwelling older adults remains unclear.

Vitamin D is a fat‐soluble secosteroid that is primarily synthesized following solar ultraviolet light exposure and is most commonly implicated in regulating bone health by increasing intestinal absorption of calcium and phosphate.^8^ Recently, vitamin D has been proposed as a modulator of skeletal muscle function, up‐regulating mitochondrial ATP production, and mitigating oxidative stress via overexpression of vitamin D receptors (VDR) in skeletal muscle stem cells.^9^, ^10^ Moreover, *in vivo* studies have linked VDR overexpression with skeletal muscle hypertrophy.^11^, ^12^ However, findings from comparable studies in older adult populations remain unequivocal.^13^ Mixed findings have been reported for associations between vitamin D status or supplementation and appendicular lean mass (ALM), timed up and go (TUG), short physical performance battery (SPPB) scores, and knee extension and handgrip strength (HGS), with some studies showing improvements,^14^, ^15^, ^16^, ^17^ but others showing negative^18^, ^19^, ^20^ or no effect.^21^ Evidence also shows that intermittent high‐dose vitamin D supplementation in individuals with adequate serum vitamin D status increases risk of falls.^22^, ^23^ Meta‐analyses have reported a small positive effect of combined vitamin D and calcium supplementation on HGS^24^, ^25^ and balance,^26^ whereas others have reported no apparent improvements in HGS and a small reduction in mobility^27^ or physical performance.^28^ Considerable heterogeneity between older adult populations (i.e. community‐dwelling vs. institutionalized) and co‐supplementation (e.g. combined vitamin D and calcium supplementation) may account for the discrepant findings between studies.

The independent effect of vitamin D supplementation on indices of sarcopenia has not been comprehensively established in community‐dwelling older adults, and so the aim of this systematic review and meta‐analysis was to investigate the effect of vitamin D supplementation as a monotherapy on muscle mass, strength, and physical performance in this population.

## Methods

This systematic review and meta‐analysis was conducted in accordance with Preferred Reporting Items for Systematic Reviews and Meta‐Analyses (PRISMA) guidelines.^29^ The protocol was registered in the International Prospective Register of Systematic Reviews (PROSPERO) (CRD: 42021240037).

### Search strategy

Two independent reviewers (K. P. and K. K. T.) searched PubMed, Embase, Web of Science, and Cochrane library from inception until March 2021. The full search strategy and the search terms used are described in the Supporting Information, *Table* S1. Only RCTs were selected, while no restrictions in terms of geographic region were applied. A manual search of references cited in the selected articles, and published reviews also were performed. Discrepancies in the literature search process were resolved by a third investigator (P. G.).

Studies were included based on the following criteria: (i) RCTs; (ii) healthy and/or community‐dwelling adults; (iii) intervention group received vitamin D supplementation as monotherapy; (iv) control group received placebo; (v) participants aged ≥50 years (there is general consensus that onset of sarcopenia begins between 50 and 60 years of age^30^). Measurements included in the European Working Group on Sarcopenia in Older People (EWGSOP2),^31^ Sarcopenia Definitions and Outcomes Consortium (SDOC),^32^ and Asian Working Group for Sarcopenia (AWGS)^33^ definitions of sarcopenia were deemed eligible for inclusion. Published articles were excluded if they (i) were reviews, letters, animal experiments, or commentaries; (ii) were not published as a full text; (iii) included participants aged <50 years; (iv) included participants with a major co‐morbidity (i.e. diabetes, cardiovascular disease, renal dysfunction, cancer, frailty, and osteoporosis) or conditions known to influence vitamin D metabolism (i.e. hyperthyroidism); (v) included institutionalized individuals; (vi) administered vitamin D supplements in the form of fortified foods; (vii) included a control group that received any form of vitamin D supplementation; (viii) used a vitamin D analogue.

### Data extraction and risk of bias

Two authors (K. P. and K. K. T.) extracted data independently which included name of first author, date of publication, country of origin, number of participants, outcome measurements, and treatment type, dose, and duration. Disagreements between authors were resolved by two independent reviewers (P. G. and K. S. K.). The quality of included studies was evaluated using the risk‐of‐bias 2 (RoB2) tool^34^ and performed by two independent reviewers (K. P. and K. K. T.). RoB2 is a comprehensive tool used to assess bias in RCTs based on the following domains: (i) randomization process; (ii) deviations from intended interventions; (iii) missing outcome data; (iv) measurement of the outcome; (v) selection of the reported result.^35^ According to the scoring system, study bias was defined as ‘high’, ‘some concerns’, or ‘low’.

### Statistical analysis

Our meta‐analysis compared changes in HGS, SPPB, TUG, ALM, and general muscle strength and physical performance in participants randomized to vitamin D supplementation or placebo.

Quantitative data were treated as continuous measurements, and changes in outcomes from baseline to follow‐up were compared between groups to calculate mean differences. When units of measurements were inconsistent and could not be converted to units required for be included in the analysis and/or outcomes measured the same aspects of muscle health, standardized mean differences were used. When numerical data were not reported, graphical values were estimated using DigitizIt 2.5 Software. Statistical significance was assessed using the random effects model and inverse‐variance method. Any missing standard deviations for changes between baseline and follow‐up among outcome measurements were estimated depending on availability of either confidence intervals, standard errors, *t* and *P* values or by calculating a correlation coefficient from a known change from baseline standard deviation derived from a similar study.

Statistical heterogeneity of outcome measurements between different studies was assessed using the overlap of their confidence interval (95% CI) and expressed as measurements of Cochran's *Q* (*χ* ^2^ test) and *I* ^2^. The classification of data as moderately heterogeneous was based on *I* ^2^ from 30 to 49% and highly heterogeneous from 50% and above.^36^ Furthermore, sensitivity analyses were performed to evaluate the robustness of reported statistical results by discounting the effect of lifestyle advice (i.e. mineral consumption and physical activity) on outcome measurements and according to risk of bias of the included studies. Subgroup analyses based on sex, treatment duration and dose of vitamin D supplementation, and geographic origin of study were also performed. The meta‐analysis was synthesized using Review Manager (RevMan 5.4.1) software.

## Results

**Figure 1:**
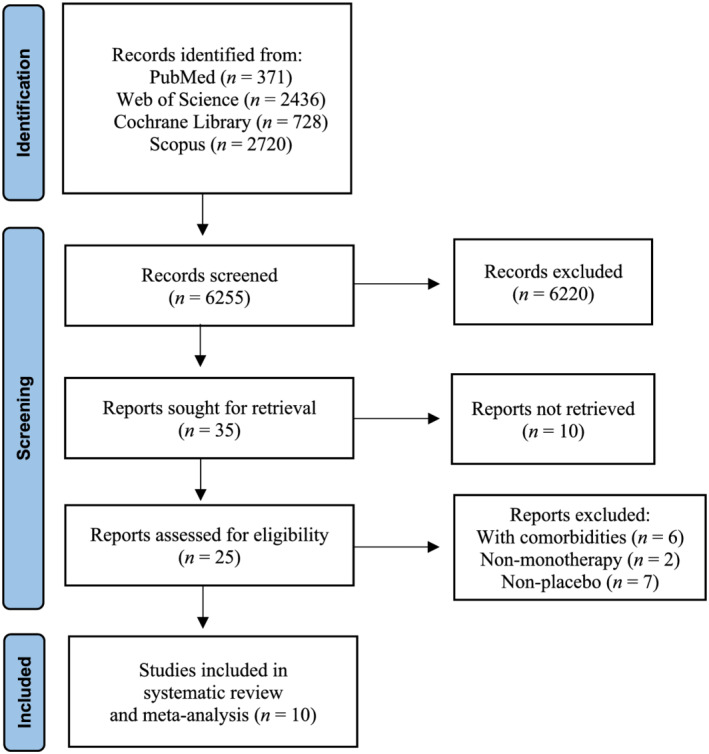
Flowchart of the search strategy employed in the literature search.

The initial literature search yielded 6255 publications. After duplicates and abstracts were excluded, 25 full‐texts were identified, and 10 studies were deemed eligible for the systematic review and meta‐analysis^37^, ^38^, ^39^, ^40^, ^41^, ^42^, ^43^, ^44^, ^45^, ^46^ (*Figure* 1). Baseline participant characteristics of the included studies are outlined in *Table* 1. Six studies were conducted in the USA,^37^, ^38^, ^41^, ^43^, ^45^, ^46^ one in Europe,^42^ and two in Australia.^40^, ^44^ Four were conducted in cohorts of both older men and women,^37^, ^38^, ^43^, ^44^ four in postmenopausal women,^39^, ^40^, ^41^, ^45^ and one in older men.^46^ In one study, the number of men and women was not reported.^42^ Vitamin D supplementation was administered as calciferol in all studies, except in that of Grady *et al*. where calcitriol was administered.^38^ Serum 25(OH)D levels were measured in all studies at follow‐up, except for Grady *et al*.^38^ where 1,25‐dihydroxyvtamin D [1,25[OH]_2_D] was measured. According to the cut off values established by the Endocrine Society,^47^ one study included participants with sufficient levels of baseline serum 25(ΟΗ)D (≥30 ng/mL),^38^ four included participants with insufficient levels (>20 and <30 ng/mL),^40^, ^41^, ^42^, ^43^ and five included participants with deficient levels (<20 ng/mL).^37^, ^39^, ^42^, ^44^, ^45^ Studies administered vitamin D supplementation daily,^37^, ^39^, ^44^, ^45^, ^46^ twice daily,^38^ weekly,^42^ twice monthly,^43^ and once every 3 months.^40^ In the remaining study, vitamin D supplementation was also administered once every 3 months, but thereafter serum 25(OH)D concentration levels were maintained between 30 and 69 ng/mL.^41^ The duration of supplementation exceeded 12 months in four studies,^37^, ^39^, ^41^, ^43^ and in the remaining six studies, the intervention was <12 months.^38^, ^40^, ^42^, ^44^, ^45^, ^46^ One study advised participants to aim to achieve daily dietary calcium consumption of 1300 mg and 30 min of physical activity.^40^

**Table 1:** Study and participant characteristics of the included studies in the meta‐analysis

Study, year	Country	Study design	Vitamin D^a^		Placebo				Treatmentdose	Treatment duration	Sarcopenia outcomes
			*n* (M/F)	Age	Baseline 25(OH)D^b^	*n* (M/F)	Age	Baseline 25(OH)D^b^			
Shea, 2019	USA	Double‐blind RCT	49 (32/17)	70.1 (±7.4)	19.6 (6.6)	51 (32/19)	69.2 (±6.2)	20.8 (6.9)	853 IU/day	12 months	HGS SPPB SCT
Aloia, 2019	USA	Double‐blind RCT	130 (0/130)	67.8 (65.1–71.5)	21.5 (6.5)	130 (0/130)	69 (65.4–73.4)	22.2 (6.9)	3490 IU/day^c^	36 months	HGS SPPB
Levis, 2016	USA	Double‐blind crossover RCT	66 (66/0)	71.8 (±6.3)	23.1 (5.0)	64 (64/0)	73.0 (±7.3)	22.6 (5.3)	4000 IU/day	9 months	HGS SPPB GST CST
Hansen, 2015	USA	Double‐blind RCT	79 (0/79)	60.0 (±5.0)	21.0 (3.0)	76 (0/76)	61.0 (±6.0)	21.0 (3.0)	50 000 IU/month	12 months	TUG ALM CST
Cangussu, 2015	Brazil	Double‐blind RCT	80 (0/80)	58.8 (±6.6)	15.0 (7.5)	80 (0/80)	59.3 (±6.7)	16.9 (6.7)	1000 IU/day	9 months	HGS CST ALM
Pirotta, 2015	Australia	Double‐blind RCT	13 (5/8)	66.1 (±4.0)	18.6 (4.6)	13 (8/5)	71.5 (±5.7)	19.4 (4.5)	2000 IU/day	10 weeks	KET TUG
Ceglia, 2013	USA	Double‐blind RCT	9 (0/9)	76.0 (± 4.0)	43.6 (10.3)	12 (0/12)	80.0 (±5.0)	48.3 (8.8)	4000 IU/day	4 months	SPPB
Glendenning, 2012	Australia	Double‐blind RCT	353 (0/353)	76.9 (± 4.0)	26.0 (7.1)	333 (0/333)	76.5 (±4.0)	26.6 (10.9)	150 000 IU/3 months	9 months	HGS TUG
Lips, 2010	Europe and North America	Double‐blind RCT	114 (NA)	78.5 (±6.2)	13.7 (4.4)	112 (NA)	77.6 (±6.6)	14.1 (5.5)	8400 IU/week	16 weeks	SPPB GST
Grady, 1991	USA	Double‐blind RCT	50 (27/23)	79.4 (±5.4)	24.2 (14.1)	48 (22/26)	78.9 (±5.4)	26.3 (20.6)	0.5 μg/day	6 months	HGS

Values are presented as mean (±standard deviation), unless otherwise stated.

25‐hydroxyvitamin D, [(25(OH)D]; ALM, appendicular lean mass; CST, chair stand test; F, female; GST, gait speed test; HGS, handgrip strength; IU, international units; KET, knee extension test at 180°; M, male; RCT, randomized controlled trial; SCT, stair climbing test; SPPB, short physical performance battery; TUG, timed up and go.

a Vitamin D supplementation was administered as calciferol in all studies, except in that of Grady *et al*.^38^ where calcitriol was used instead.

b Baseline serum 25‐hydroxyvitamin D 25(OH)D concentrations were expressed as ng/mL.

c Doses were adjusted in 3‐month intervals to maintain serum 25(OH)D concentrations between 30 and 69 ng/mL.

### Assessed indices of sarcopenia

Handgrip strength was expressed in kilograms (kg) or pounds/square inch (lb/in^2^) and assessed with the use of hydraulic or pneumatic hand dynamometers.^31^ ALM was estimated by dual‐energy X‐ray absorptiometry (DXA), was defined as the sum of lean tissue in both upper and lower limbs, and was expressed in kilograms/squared meters (kg/m^2^).^48^ SPPB score was calculated based on individual scores for balance, gait speed, and a chair stand test. The balance test was measured with the participant being asked to hold several standing positions, including a side‐by‐side, a semi‐tandem, and a tandem position for 10 s.^49^ Gait speed was assessed through a 4 m walking speed test, measured either with a stopwatch or an electronic device.^50^ Finally, the chair stand test was evaluated by the duration needed for a participant to rise five times from a seated position without using their arms.^51^ Each test was scored on a 0–4 scale for a total SPPB score of 0–12.^52^ TUG was defined by the time taken to rise from a chair, walk 3 m, turn around 180°, and walk back to sit on the chair.^53^ TUG was expressed in seconds. Chair stand test (CST) was evaluated by the number of repetitions a patient could rise from and sit in a chair within 30 s.^54^ Leg strength was assessed via a knee extension exercise in which participants were asked to extend their knees at a 120‐degree angle.^55^ The stair climbing test (SCT) involved timing the duration of climbing a flight of 10 stairs using a stopwatch.^56^ General muscle strength was expressed as a composite plot of any muscle strength measures (HGS and knee extension at 180°), and general physical performance was a composite plot that included any measure of physical performance (SPPB, TUG, or CST). Serum 25‐hydroxyvitamin D [25(OH)D] concentration was expressed in nanograms/millilitre (ng/mL) and serum 1,25(OH)_2_D was expressed in pmol/litre (pmol/L). When expressed in nmol/L, serum 25(OH)D concentration was converted to ng/mL using a factor of 2.5.^57^ When a study administered multiple treatment arms with different vitamin D supplementation doses, only the arm with the highest dose was considered.

### Risk of bias of included studies

Two studies had some concerns in terms of the randomization process as they did not report relevant information on this process (*Figure* S1).^41^, ^46^ One study had high risk of bias regarding the randomization process because there was no information about concealment of treatment allocation.^38^ One study had high risk of bias arising from missing outcome data.^38^ One study had some concerns in the measurement of outcomes due to lack of relevant information on the assessment process.^46^ One study had some concerns of bias in the selection of the reported results arising from absence of the data analysis procedure employed.^38^

### Vitamin D supplementation and indices of sarcopenia

**Figure 2:**
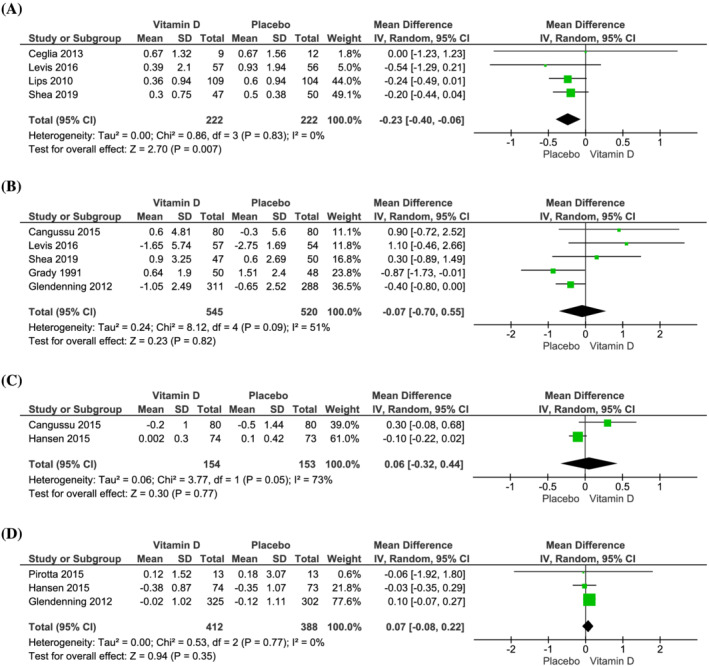
Effect of vitamin D supplementation on changes in (*A*) short physical performance battery, (*B*) handgrip strength, (*C*) appendicular lean mass, and (*D*) timed up and go, compared with placebo.

**Figure 3:**
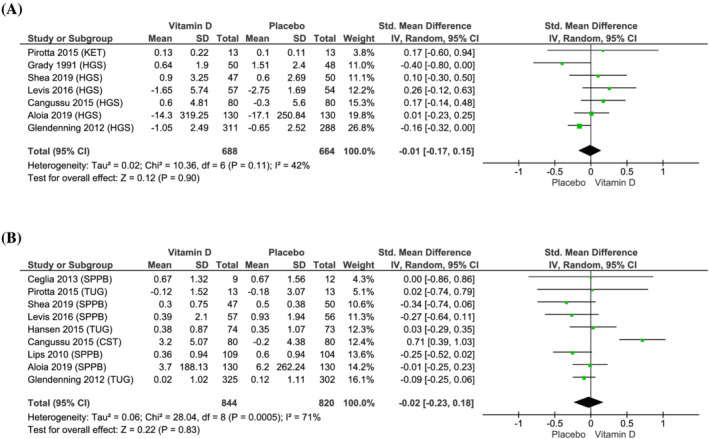
Effect of vitamin D supplementation on changes in (*A*) general muscle strength [handgrip strength (HGS) and knee extension test at 180° (KET)] and (*B*) general physical performance [(short physical performance battery (SPPB), timed up and go (TUG) and chair stand test (CST)], compared with placebo.

Serum vitamin D [25(OH)D and 1,25(OH)_2_D] concentrations increased following vitamin D treatment compared to placebo (SMD: 1.97; 95% CI: 1.30–2.64; *I* ^*2*^ = 95%, *P* < 0.00001) (*Figure* S2). Our analysis revealed that vitamin D supplementation decreased SPPB scores compared with placebo (MD: −0.23; 95% CI −0.40 to −0.06; *I* ^*2*^ = 0%; *P* = 0.007) (*Figure* 2A). Vitamin D supplementation had no effect on HGS compared with placebo (MD: −0.07 kg; 95% CI −0.70 to 0.55; *I* ^*2*^ = 51%, *P* = 0.82) (*Figure* 2B). Changes in other outcome measurements of sarcopenia were also similar between groups, specifically ALM (MD: 0.06 kg/m^2^; 95% CI: −0.32 to 0.44; *I* ^*2*^ = 73%, *P* = 0.77) (*Figure* 2C), TUG (MD: 0.07 s; 95% CI −0.08 to 0.22; *I* ^*2*^ = 0%, *P* = 0.35) (*Figure* 2D), general muscle strength (SMD: −0.01; 95% CI −0.17 to 0.15; *I* ^*2*^ = 42%, *P* = 0.90) (*Figure* 3A), and general physical performance (SMD: −0.02; 95% CI −0.23 to 0.18; *I* ^*2*^ = 71%, *P* = 0.83) (*Figure* 3B).

A series of subgroup analyses based on sex (women and men; women only), treatment duration (<6 months; >6 months), and dose of vitamin D supplementation (moderate: 853–1667 IU/day), and geographic origin of study (USA; rest of the world) was performed for HGS, and the effect remained unchanged (*Figure* S3A–D). No effect of vitamin D supplementation on HGS was observed for women and men (MD: 0.04 kg; 95% CI: −1.10 to 1.19; *I* ^*2*^ = 65%, *P* = 0.94) or women alone (MD: 0.00 kg; 95% CI: −1.18 to 1.18, *I* ^*2*^ = 57%, *P* = 0.99), or in studies performed in the USA (MD: 0.04 kg, 95% CI: −1.10 to 1.19, *I* ^*2*^ = 65%, *P* = 0.94) or the rest of the world (MD: 0.00 kg, 95% CI: −1.18 to 1.18, *I* ^*2*^ = 57%, *P* = 0.99). Studies administering moderate doses of vitamin D supplementation also had no effect on HGS (MD: −0.03 kg, 95% CI: −0.74 to 0.68, *I* ^*2*^ = 39%, *P* = 0.93). No effect of vitamin D supplementation on HGS was observed for studies with treatment duration of more than 6 months (MD: 0.67 kg, 95% CI: −0.14 to 1.49, *I* ^*2*^ = 0%, *P* = 0.11), but treatment duration of <6 months had a significant negative effect on HGS (MD: −0.48 kg, 95% CI: −0.85 to −0.12, *I* ^*2*^ = 0%, *P* = 0.009),

Lifestyle factors also had no effect on our findings (*Figure* S4A,B); after omission of studies that provided advice on calcium consumption and physical activity, changes in HGS (MD: 0.20 kg, 95% CI: −0.78 to 1.19, *I* ^*2*^ = 59%, *P* = 0.68) and serum vitamin D [25(OH)D and 1,25(OH)_2_D] concentrations (SMD: 2.13, 95% CI: 1.42–2.84, *I* ^*2*^ = 95%, *P* < 0.00001) were similar to those observed in primary analyses. Type of vitamin D supplementation and study risk of bias also had no effect on our findings (*Figure* S5A–C); results were similar after omission of studies that administered calcitriol as a treatment or that were of high risk of bias for changes in HGS (MD: 0.22 kg, 95% CI: −0.56 to 0.99, *I* ^*2*^ = 51%, *P* = 0.59), global muscle strength (SMD: 0.02, 95% CI: −0.12 to 0.17, *I* ^*2*^ = 30%, *P* = 0.76), and serum vitamin D [25(OH)D and 1,25(OH)_2_D] concentrations (SMD: 2.17, 95% CI: 1.54–2.81, *I* ^2^ = 94%, *P* < 0.00001).

## Discussion

This systematic review and meta‐analysis examined the effects of vitamin D supplementation as a monotherapy, compared to placebo, on muscle mass, strength and physical performance in community‐dwelling older adults. Vitamin D supplementation decreased SPPB scores (indicating negative effects on physical performance) but had no effect on muscle mass, strength, or any other physical performance parameters. These observations were based on findings from 10 studies with a low overall risk of bias.

Vitamin D supplementation led to a significant reduction in SPPB scores among community‐dwelling older adults compared to placebo. Perera *et al*. have proposed meaningful decline estimates of 0.27–0.55 for SPPB score, which suggests that the between‐group difference in change in SPPB score of −0.23 from baseline to follow‐up we observed for vitamin D compared with placebo could be clinically relevant.^58^ However, further research is required to confirm the meaningfulness of a difference in SPPB change of this magnitude, and it was not possible to explore how individual indices of SPPB (such as balance) changed in response to vitamin D supplementation.^58^ Furthermore, in two studies,^37^, ^41^ participants had perfect SPPB scores at baseline, resulting in a ceiling constraint on the effect of vitamin D supplementation. Nevertheless, previous studies have reported that high‐dose vitamin D supplementation increases risk of falls,^22^, ^59^ which may occur via supplement‐related decreases in physical performance. To explore this potential mechanism, we compared changes in general physical performance, which included any measure of physical performance to increase statistical power, but we found no differences between groups. This could be due to our inability to include only studies that prescribed high‐dose vitamin D supplementation in these analyses (insufficient number of studies), and so further research is required to explore how high‐dose vitamin D supplementation influences physical performance in older populations. However, based on current evidence suggesting increased risk of falls, mechanistic studies may be preferable to investigate this relationship and any randomized controlled trials of high‐dose vitamin D supplementation should potentially be restricted to those at low risk of falling.

The age‐related decline in muscle mass and strength may place older adults at an increased risk of falls and fractures.^60^ Therefore, sustaining mobility during ageing is critical to reduce the risk of falls and fractures, as well as subsequent immobilization following a fall/fracture that may accelerate the incidence of sarcopenia. In this meta‐analysis, TUG, an indicator of mobility levels in older adults, demonstrated no changes in response to vitamin D supplementation. Consistent with this observation, other meta‐analyses have reported no differences in mobility levels after daily vitamin D administration with 1000 IU,^25^, ^61^ while two other meta‐analyses found a minor increase in TUG times (indicating worsening mobility) following vitamin D supplementation.^27^, ^28^

Vitamin D supplementation had no effect on HGS compared to placebo, although negative effects of treatment durations <6 months were observed. HGS has been proposed as a valuable and reliable assessment of muscle strength due to its low cost and practicability in clinical and community healthcare settings.^62^ A previous meta‐analysis reported minor improvements in HGS following 1000 IU/day of vitamin D supplementation, and greater benefits were observed in older adults aged ≥65 years.^24^ Another meta‐analysis reported improvements in several independent measurements of muscle strength (handgrip, quadriceps, and knee extension strength) following combined vitamin D and calcium supplementation,^26^ while Beaudart *et al*. revealed a small but significant effect of vitamin D supplementation on general muscle strength, when combining multiple measures of muscle strength.^24^ Conversely, a recent meta‐analysis reported no effect of vitamin D supplementation on HGS or SPPB in older adults compared with placebo.^28^ Other meta‐analyses have also reported that vitamin D supplementation has no effect on HGS in community‐dwelling or pre‐frail older adults.^15^, ^27^ However, these meta‐analyses included studies that combined vitamin D and calcium supplementation,^15^, ^27^ as well as studies that recruited individuals with co‐morbidities (i.e. type 2 diabetes, hyperparathyroidism, and chronic obstructive pulmonary disease) that known to affect vitamin D absorption kinetics.^63^, ^64^, ^65^ To the best of our knowledge, ours is the first meta‐analysis to demonstrate a negative effect of vitamin D monotherapy on hand grip strength when administered for <6 months. While it should be noted that this observation was obtained from a sub‐group analysis of only two studies, Sanders *et al*. previously reported that the risk of falls and fracture in older women receiving intermittent annual high‐dose (500 000 IU) vitamin D monotherapy increased in the first 3 months following vitamin D supplementation.^22^ The authors proposed that this effect on falls could be explained by the intermittent, high‐dosing regimen; however, the results of our meta‐analysis suggest that further research is warranted to explore short‐term effects of different vitamin D supplementation regimens on muscle function.

According to the EWGSOP and AWGS definitions, the evaluation of ALM as a surrogate measurement of muscle mass constitutes a valuable parameter for the diagnosis of sarcopenia.^31^, ^33^ In our meta‐analysis, no effect of vitamin D supplementation on ALM was observed, although the analysis included only two studies.^39^, ^43^ Prior meta‐analyses in older adults reported a modest increase in ALM following vitamin D supplementation.^24^, ^66^ However, one meta‐analysis investigated the combined use of vitamin D with protein supplementation in healthy older adults,^66^ whereas another meta‐analysis used total lean body mass, rather than ALM, for the assessment of muscle mass using DXA.^24^ Protein supplementation alone is an effective nutritional strategy to increase muscle mass in older adults,^67^ and total lean body mass may be more influenced by inclusion of non‐muscle tissues than ALM.^68^ Consistent with our findings, a more recent meta‐analysis reported no beneficial effect of vitamin D supplementation on ALM in older adults.^28^ Hence, findings from past and present studies appear to be inconsistent regarding the effects of vitamin D supplementation on muscle mass in older populations. Thus, future trials are warranted to explore the effect of vitamin D supplementation on muscle mass in community‐dwelling older adults.

### Strengths and limitations

This is the first study to examine the effect of vitamin D supplementation as a monotherapy on indices of sarcopenia (measures of muscle mass, strength, and physical performance) in community‐dwelling older adults, compared with placebo. Previous studies have only investigated vitamin D effects without controlling for confounders such as calcium co‐supplementation and co‐morbidity status. Increased calcium intake has been associated with lower odds of sarcopenia,^69^, ^70^ elevated ALM,^69^, ^70^ and greater gait speed.^71^ Equally, an association with attenuated physical function and lower muscle quality have all been observed in individuals with obesity and related co‐morbidities.^72^, ^73^, ^74^ This suggests that mineral co‐supplementation and co‐morbidity status may potentially influence effects of vitamin D supplementation on muscle health. Moreover, to achieve the aim of our study, we utilized clinically reliable measurements of physical performance and muscle strength that have little bias in their determination among older populations. However, our study is limited by the scarcity of similar studies in terms of sex ratios, vitamin D treatment regimes, serum 25(OH)D baseline values, and assessments of sarcopenia indices. Additionally, effects of lower (i.e. <400 IU/day) and higher (i.e. >4000 IU/day) dose vitamin D supplementation could not be explored. Further, daily physical activity in the included studies was not accounted for in a way that ensured comparability of post‐intervention sarcopenia indices between the two groups. Finally, in two studies with substantial population weights in our analysis,^38^, ^46^ signs of high risk or some concerns in terms of bias were observed. Taken together, our findings should be interpreted with caution, and their clinical meaningfulness warrants further investigation.

## Conclusions

This systematic review and meta‐analysis revealed no effect of vitamin D supplementation on muscle mass or strength, but resulted in a significant decrease in SPPB score, indicating potential deleterious effects on lower‐limb physical function, when administered as a monotherapy in community‐dwelling older adults. A sub‐group analysis also revealed decreases in HGS following vitamin D supplementation regimens of <6 months. Future studies that supplement vitamin D in populations with low vitamin D status and/or low baseline physical performance are warranted to provide insight on its effects on sarcopenia indices in these groups, particularly for balance and mobility measures which comprise the SPPB. Further research is also necessary to confirm effects of short‐term vitamin D supplementation on muscle function in older adults. The lack of homogeneity in sarcopenia indices highlights the need for a global consensus regarding clinically relevant measurement tools for assessing muscle mass, strength, and physical performance in older populations. However, based on current evidence, the impact of vitamin D supplementation in mitigating risk of sarcopenia may be considered negligible, at least in community‐dwelling older adults.

## Conflict of interest

There are no conflict of interest.

## Supporting information




**Table S1.** Search terms employed to screen different electronic databases for the literature search.

**Figure S1.** Quality assessment of the included studies according to the Cochrane risk‐of‐bias 2 tool.
**Figure S2.** Effect of vitamin D supplementation on changes in serum vitamin D (25‐hydroxyvitamin D [25(OH)D] and 1,25‐dihydroxyvitamin D [1,25(OH)2D]) levels compared to placebo.
**Figure S3.** Subgroup analysis of handgrip strength changes in response to vitamin D supplementation based on **(A)** sex, vitamin D treatment **(B)** duration and **(C)** dose and **(D)** geographic origin of study, compared to placebo.
**Figure S4.** Sensitivity analysis based on the effect of lifestyle factors on changes in **(A)** handgrip strength **(B)** serum vitamin D (25‐hydroxyvitamin D [25(OH)D] and 1,25‐dihydroxyvitamin D [1,25(OH)2D]), in response to vitamin D supplementation compared to placebo.
**Figure S5.** Sensitivity analysis based on the effect of type of vitamin D supplementation or risk of bias on changes in **(A)** handgrip strength (HGS), **(B)** general muscle strength (HGS and knee extension test at 180 degrees (KET)) and **(C)** serum vitamin D (25‐hydroxyvitamin D [25(OH)D] and 1,25‐dihydroxyvitamin D [1,25(OH)2D]) levels, in response to vitamin D supplementation compared to placebo.
