# Supplementary material for: Effect of vitamin D monotherapy on indices of sarcopenia in community‐dwelling older adults: a systematic review and meta‐analysis
Source: J Cachexia Sarcopenia Muscle. 2022 Mar 8;13(3):1642–52. doi: 10.1002/jcsm.12976 (PMC9178168; doi:10.1002/jcsm.12976)

Supplementary Figures

Figure S1. Quality assessment of the included studies according to the Cochrane risk-of-bias 2 tool.

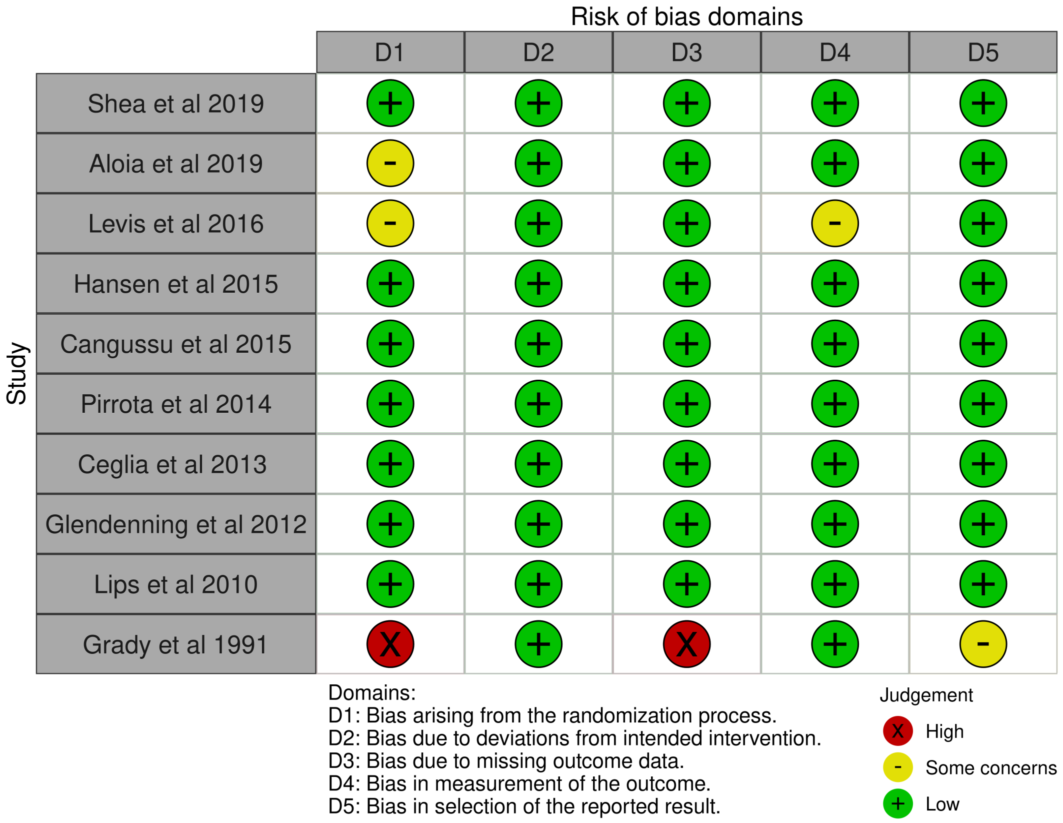

Figure S2. Effect of vitamin D supplementation on changes in serum vitamin D (25-hydroxyvitamin D [25(OH)D] and 1,25-dihydroxyvitamin D [1,25$(OH)_2$D]) levels compared to placebo.

	Vitamin D			Placebo				Std. Mean Difference
Study or Subgroup	Mean	SD	Total	Mean	SD	Total	Weight	IV, Random, 95% CI
Pirotta 2015 [25(OH)D]	13.6	4.14	13	-0.48	2.39	13	7.4%	4.03 [2.62, 5.45]
Ceglia 2013 [25(OH)D]	14.56	6.44	9	1.68	5.52	12	8.5%	2.09 [0.97, 3.20]
Shea 2019 [25(OH)D]	13.2	4.97	47	-1	2.78	48	10.0%	3.51 [2.86, 4.16]
Glendenning 2012 [25(OH)D]	3.84	13.04	20	-2.52	9.61	20	10.1%	0.54 [-0.09, 1.18]
Hansen 2015 [25(OH)D]	21	8.78	79	-3	4.86	76	10.4%	3.35 [2.86, 3.84]
Levis 2016 [25(OH)D]	23.02	14.19	56	1.18	5.83	55	10.5%	1.99 [1.54, 2.45]
Grady 1991 [1,25(OH)D]	9.9	46.19	50	2.7	25.43	48	10.7%	0.19 [-0.21, 0.59]
Lips 2010 [25(OH)D]	26.4	7	99	13.5	7.7	97	10.8%	1.75 [1.42, 2.08]
Cangussu 2015 [25(OH)D]	12.48	23.54	80	-3.14	9.92	80	10.8%	0.86 [0.54, 1.18]
Aloia 2019 [25(OH)D]	25.3	15.23	130	-1.8	9.9	130	10.8%	2.10 [1.80, 2.41]
**Total (95% CI)**			**583**			**579**	**100.0%**	**1.97 [1.30, 2.64]**

Heterogeneity: Tau² = 1.06; Chi² = 186.19, df = 9 (P < 0.00001); I² = 95%
Test for overall effect: Z = 5.75 (P < 0.00001)

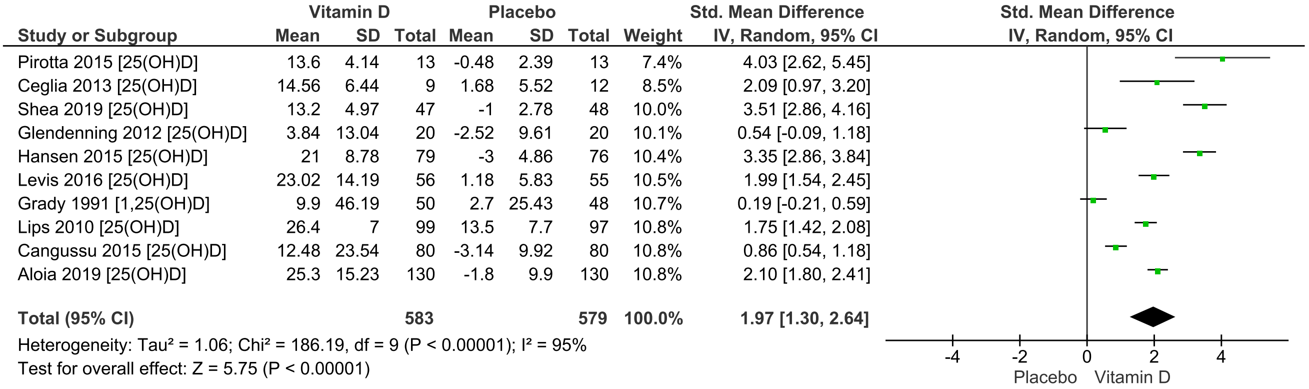

Figure S3. Subgroup analysis of handgrip strength changes in response to vitamin D supplementation based on **(A)** sex, vitamin D treatment **(B)** duration and **(C)** dose and **(D)** geographic origin of study, compared to placebo.

A

Study or Subgroup	Vitamin D Mean	Vitamin D SD	Vitamin D Total	Placebo Mean	Placebo SD	Placebo Total	Weight	Mean Difference IV, Random, 95% CI
**Women and men**								
Levis 2016	-1.65	5.74	57	-2.75	1.69	54	11.8%	1.10 [-0.46, 2.66]
Shea 2019	0.9	3.25	47	0.6	2.69	50	16.8%	0.30 [-0.89, 1.49]
Grady 1991	0.64	1.9	50	1.51	2.4	48	23.8%	-0.87 [-1.73, -0.01]
**Subtotal (95% CI)**			**154**			**153**	**52.4%**	**0.04 [-1.10, 1.19]**
Heterogeneity: Tau² = 0.66; Chi² = 5.69, df = 2 (P = 0.06); I² = 65%								
Test for overall effect: Z = 0.07 (P = 0.94)								
**Women**								
Cangussu 2015	0.6	4.81	80	-0.3	5.6	80	11.1%	0.90 [-0.72, 2.52]
Glendenning 2012	-1.05	2.49	311	-0.65	2.52	288	36.5%	-0.40 [-0.80, 0.00]
**Subtotal (95% CI)**			**391**			**368**	**47.6%**	**0.00 [-1.18, 1.18]**
Heterogeneity: Tau² = 0.48; Chi² = 2.34, df = 1 (P = 0.13); I² = 57%								
Test for overall effect: Z = 0.01 (P = 0.99)								
**Total (95% CI)**			**545**			**521**	**100.0%**	**-0.07 [-0.70, 0.55]**
Heterogeneity: Tau² = 0.24; Chi² = 8.13, df = 4 (P = 0.09); I² = 51%								
Test for overall effect: Z = 0.23 (P = 0.82)								
Test for subgroup differences: Chi² = 0.00, df = 1 (P = 0.96), I² = 0%								

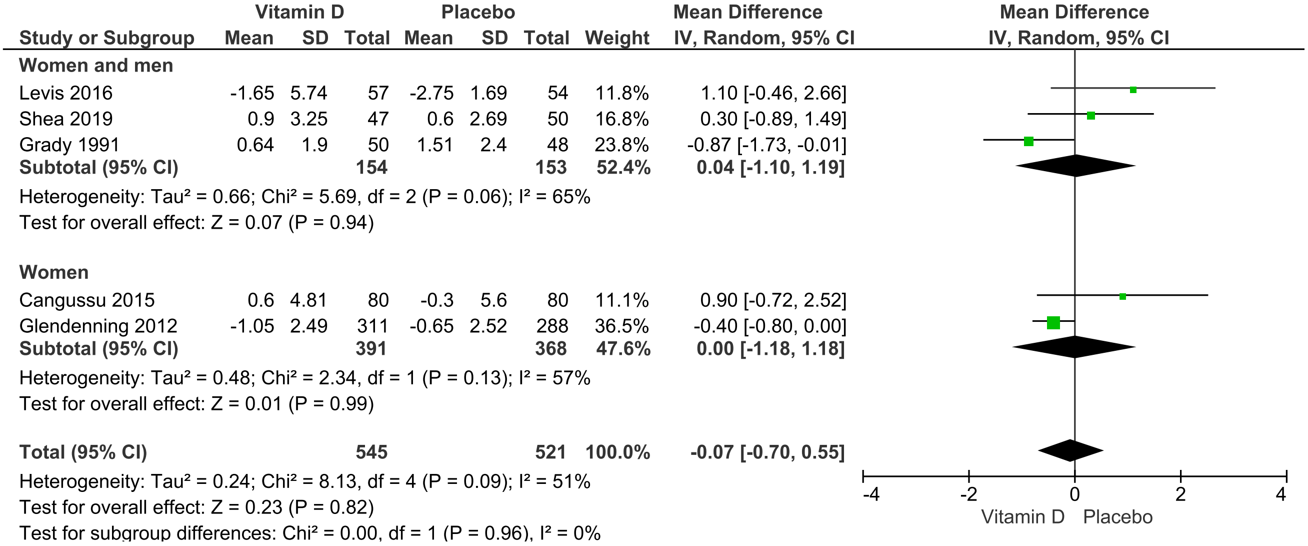

B

Study or Subgroup	Vitamin D Mean	Vitamin D SD	Vitamin D Total	Placebo Mean	Placebo SD	Placebo Total	Weight	Mean Difference IV, Random, 95% CI
**Less than 6 months**								
Grady 1991	0.64	1.9	50	1.51	2.4	48	23.8%	-0.87 [-1.73, -0.01]
Glendenning 2012	-1.05	2.49	311	-0.65	2.52	288	36.5%	-0.40 [-0.80, 0.00]
**Subtotal (95% CI)**			**361**			**336**	**60.3%**	**-0.48 [-0.85, -0.12]**
Heterogeneity: Tau² = 0.00; Chi² = 0.94, df = 1 (P = 0.33); I² = 0%								
Test for overall effect: Z = 2.61 (P = 0.009)								
**More than 6 months**								
Cangussu 2015	0.6	4.81	80	-0.3	5.6	80	11.1%	0.90 [-0.72, 2.52]
Levis 2016	-1.65	5.74	57	-2.75	1.69	54	11.8%	1.10 [-0.46, 2.66]
Shea 2019	0.9	3.25	47	0.6	2.69	50	16.8%	0.30 [-0.89, 1.49]
**Subtotal (95% CI)**			**184**			**185**	**39.7%**	**0.67 [-0.14, 1.49]**
Heterogeneity: Tau² = 0.00; Chi² = 0.74, df = 2 (P = 0.69); I² = 0%								
Test for overall effect: Z = 1.62 (P = 0.11)								
**Total (95% CI)**			**545**			**521**	**100.0%**	**-0.07 [-0.70, 0.55]**
Heterogeneity: Tau² = 0.24; Chi² = 8.13, df = 4 (P = 0.09); I² = 51%								
Test for overall effect: Z = 0.23 (P = 0.82)								
Test for subgroup differences: Chi² = 6.44, df = 1 (P = 0.01), I² = 84.5%								

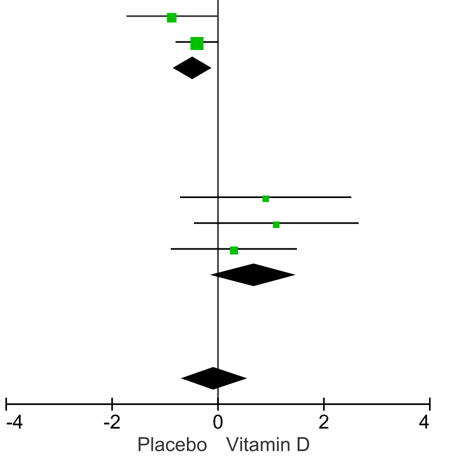

C

Study or Subgroup	Vitamin D Mean	Vitamin D SD	Vitamin D Total	Placebo Mean	Placebo SD	Placebo Total	Weight	Mean Difference IV, Random, 95% CI
**Moderate dose (853 - 1667 IU/d)**								
Cangussu 2015	0.6	4.81	80	-0.3	5.6	80	15.3%	0.90 [-0.72, 2.52]
Shea 2019	0.9	3.25	47	0.6	2.69	50	24.1%	0.30 [-0.89, 1.49]
Glendenning 2012	-1.05	2.49	311	-0.65	2.52	288	60.6%	-0.40 [-0.80, 0.00]
**Subtotal (95% CI)**			**438**			**418**	**100.0%**	**-0.03 [-0.74, 0.68]**
Heterogeneity: Tau² = 0.17; Chi² = 3.29, df = 2 (P = 0.19); I² = 39%								
Test for overall effect: Z = 0.09 (P = 0.93)								
**Total (95% CI)**			**438**			**418**	**100.0%**	**-0.03 [-0.74, 0.68]**
Heterogeneity: Tau² = 0.17; Chi² = 3.29, df = 2 (P = 0.19); I² = 39%								
Test for overall effect: Z = 0.09 (P = 0.93)								
Test for subgroup differences: Not applicable								

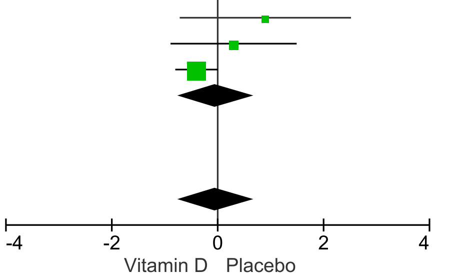

D

Study or Subgroup	Vitamin D Mean	Vitamin D SD	Vitamin D Total	Placebo Mean	Placebo SD	Placebo Total	Weight	Mean Difference IV, Random, 95% CI
**United States of America**								
Levis 2016	-1.65	5.74	57	-2.75	1.69	54	11.8%	1.10 [-0.46, 2.66]
Shea 2019	0.9	3.25	47	0.6	2.69	50	16.8%	0.30 [-0.89, 1.49]
Grady 1991	0.64	1.9	50	1.51	2.4	48	23.8%	-0.87 [-1.73, -0.01]
**Subtotal (95% CI)**			**154**			**153**	**52.4%**	**0.04 [-1.10, 1.19]**
Heterogeneity: Tau² = 0.66; Chi² = 5.69, df = 2 (P = 0.06); I² = 65%								
Test for overall effect: Z = 0.07 (P = 0.94)								
**Rest of the world**								
Cangussu 2015	0.6	4.81	80	-0.3	5.6	80	11.1%	0.90 [-0.72, 2.52]
Glendenning 2012	-1.05	2.49	311	-0.65	2.52	288	36.5%	-0.40 [-0.80, 0.00]
**Subtotal (95% CI)**			**391**			**368**	**47.6%**	**0.00 [-1.18, 1.18]**
Heterogeneity: Tau² = 0.48; Chi² = 2.34, df = 1 (P = 0.13); I² = 57%								
Test for overall effect: Z = 0.01 (P = 0.99)								
**Total (95% CI)**			**545**			**521**	**100.0%**	**-0.07 [-0.70, 0.55]**
Heterogeneity: Tau² = 0.24; Chi² = 8.13, df = 4 (P = 0.09); I² = 51%								
Test for overall effect: Z = 0.23 (P = 0.82)								
Test for subgroup differences: Chi² = 0.00, df = 1 (P = 0.96), I² = 0%								

Mean Difference
IV, Random, 95% CI

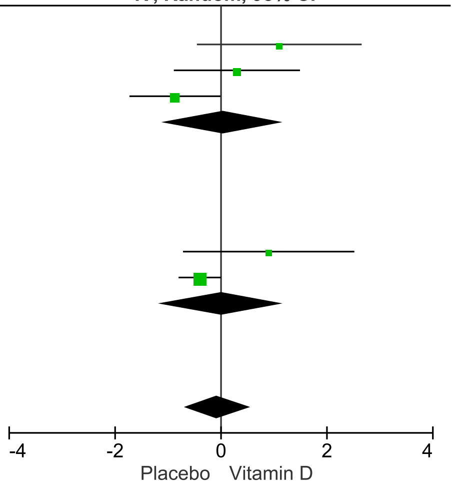

Figure S4. Sensitivity analysis based on the effect of lifestyle factors on changes in **(A)** handgrip strength **(B)** serum vitamin D (25-hydroxyvitamin D [25(OH)D] and 1,25-dihydroxyvitamin D [1,25$(OH)_2$D]), in response to vitamin D supplementation compared to placebo.

A

Study or Subgroup	Vitamin D Mean	Vitamin D SD	Vitamin D Total	Placebo Mean	Placebo SD	Placebo Total	Weight	Mean Difference IV, Random, 95% CI
**Without advice on calcium consumption and physical activity**								
Cangussu 2015	0.6	4.81	80	-0.3	5.6	80	11.1%	0.90 [-0.72, 2.52]
Levis 2016	-1.65	5.74	57	-2.75	1.69	54	11.8%	1.10 [-0.46, 2.66]
Shea 2019	0.9	3.25	47	0.6	2.69	50	16.8%	0.30 [-0.89, 1.49]
Grady 1991	0.64	1.9	50	1.51	2.4	48	23.8%	-0.87 [-1.73, -0.01]
**Subtotal (95% CI)**			**234**			**233**	**63.5%**	**0.20 [-0.78, 1.19]**
Heterogeneity: Tau² = 0.57; Chi² = 7.25, df = 3 (P = 0.06); I² = 59%								
Test for overall effect: Z = 0.41 (P = 0.68)								
**With advice on calcium consumption and physical activity**								
Glendenning 2012	-1.05	2.49	311	-0.65	2.52	288	36.5%	-0.40 [-0.80, 0.00]
**Subtotal (95% CI)**			**311**			**288**	**36.5%**	**-0.40 [-0.80, 0.00]**
Heterogeneity: Not applicable								
Test for overall effect: Z = 1.95 (P = 0.05)								
**Total (95% CI)**			**545**			**521**	**100.0%**	**-0.07 [-0.70, 0.55]**
Heterogeneity: Tau² = 0.24; Chi² = 8.13, df = 4 (P = 0.09); I² = 51%								
Test for overall effect: Z = 0.23 (P = 0.82)								
Test for subgroup differences: Chi² = 1.25, df = 1 (P = 0.26), I² = 19.7%								

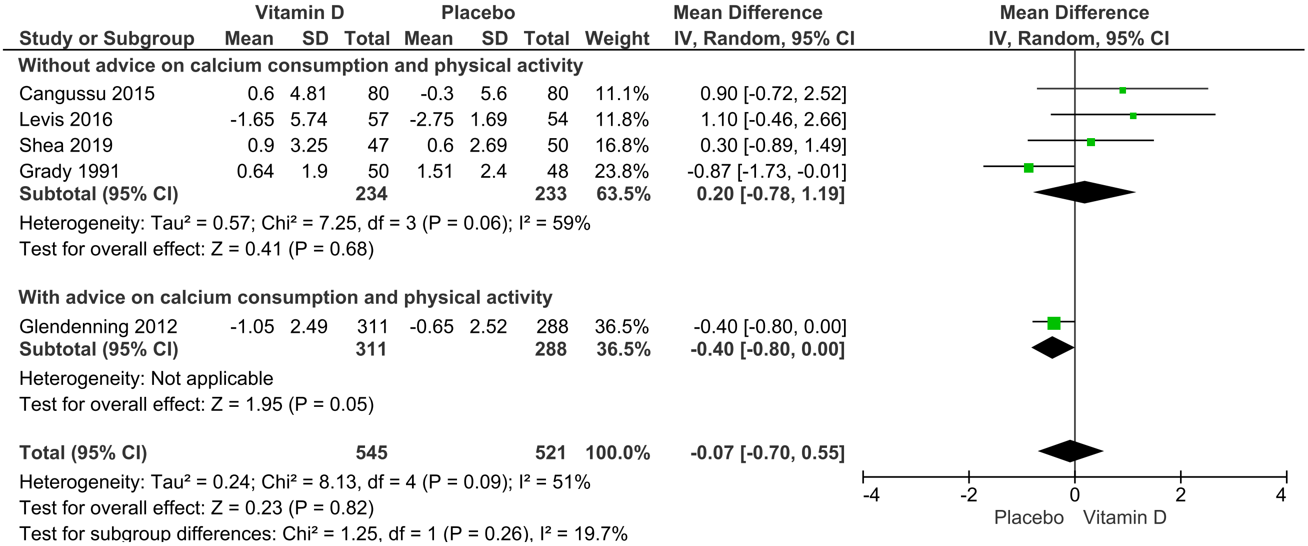

B

Study or Subgroup	Vitamin D Mean	Vitamin D SD	Vitamin D Total	Placebo Mean	Placebo SD	Placebo Total	Weight	Std. Mean Difference IV, Random, 95% CI
**Without advice on calcium consumption and physical activity**								
Pirotta 2015 [25(OH)D]	13.6	4.14	13	-0.48	2.39	13	7.4%	4.03 [2.62, 5.45]
Ceglia 2013 [25(OH)D]	14.56	6.44	9	1.68	5.52	12	8.5%	2.09 [0.97, 3.20]
Shea 2019 [25(OH)D]	13.2	4.97	47	-1	2.78	48	10.0%	3.51 [2.86, 4.16]
Hansen 2015 [25(OH)D]	21	8.78	79	-3	4.86	76	10.4%	3.35 [2.86, 3.84]
Levis 2016 [25(OH)D]	23.02	14.19	56	1.18	5.83	55	10.5%	1.99 [1.54, 2.45]
Grady 1991 [1,25(OH)D]	9.9	46.19	50	2.7	25.43	48	10.7%	0.19 [-0.21, 0.59]
Lips 2010 [25(OH)D]	26.4	7	99	13.5	7.7	97	10.8%	1.75 [1.42, 2.08]
Cangussu 2015 [25(OH)D]	12.48	23.54	80	-3.14	9.92	80	10.8%	0.86 [0.54, 1.18]
Aloia 2019 [25(OH)D]	25.3	15.23	130	-1.8	9.9	130	10.8%	2.10 [1.80, 2.41]
**Subtotal (95% CI)**			**563**			**559**	**89.9%**	**2.13 [1.42, 2.84]**
Heterogeneity: Tau² = 1.06; Chi² = 173.45, df = 8 (P < 0.00001); I² = 95%								
Test for overall effect: Z = 5.90 (P < 0.00001)								
**With advice on calcium consumption and physical activity**								
Glendenning 2012 [25(OH)D]	3.84	13.04	20	-2.52	9.61	20	10.1%	0.54 [-0.09, 1.18]
**Subtotal (95% CI)**			**20**			**20**	**10.1%**	**0.54 [-0.09, 1.18]**
Heterogeneity: Not applicable								
Test for overall effect: Z = 1.69 (P = 0.09)								
**Total (95% CI)**			**583**			**579**	**100.0%**	**1.97 [1.30, 2.64]**
Heterogeneity: Tau² = 1.06; Chi² = 186.19, df = 9 (P < 0.00001); I² = 95%								
Test for overall effect: Z = 5.75 (P < 0.00001)								
Test for subgroup differences: Chi² = 10.73, df = 1 (P = 0.001), I² = 90.7%								

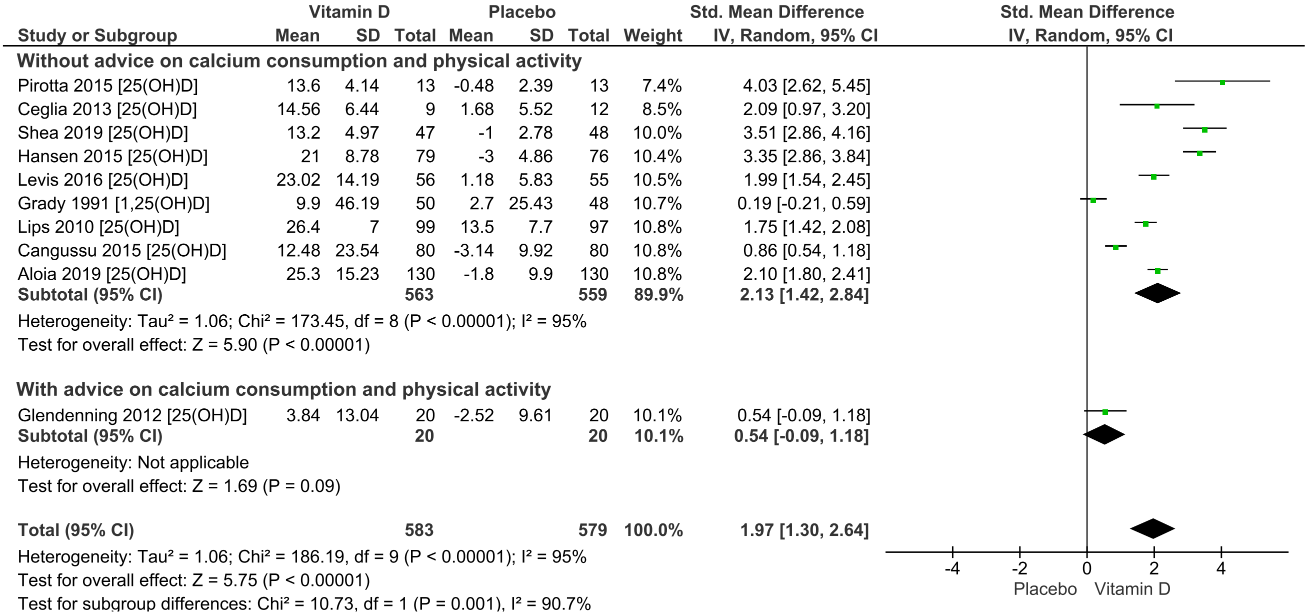

Figure S5. Sensitivity analysis based on the effect of type of vitamin D supplementation or risk of bias on changes in **(A)** handgrip strength (HGS), **(B)** general muscle strength (HGS and knee extension test at 180 degrees (KET)) and **(C)** serum vitamin D (25-hydroxyvitamin D [25(OH)D] and 1,25-dihydroxyvitamin D [1,25$(OH)_2$D]) levels, in response to vitamin D supplementation compared to placebo.

A

	Vitamin D			Placebo				Mean Difference
**Study or Subgroup**	**Mean**	**SD**	**Total**	**Mean**	**SD**	**Total**	**Weight**	**IV, Random, 95% CI**
**Calciferol supplementation / Low, some concerns risk of bias**								
Cangussu 2015	0.6	4.81	80	-0.3	5.6	80	11.1%	0.90 [-0.72, 2.52]
Levis 2016	-1.65	5.74	57	-2.75	1.69	54	11.8%	1.10 [-0.46, 2.66]
Shea 2019	0.9	3.25	47	0.6	2.69	50	16.8%	0.30 [-0.89, 1.49]
Glendenning 2012	-1.05	2.49	311	-0.65	2.52	288	36.5%	-0.40 [-0.80, 0.00]
**Subtotal (95% CI)**			**495**			**472**	**76.2%**	**0.22 [-0.56, 0.99]**
Heterogeneity: Tau² = 0.31; Chi² = 6.08, df = 3 (P = 0.11); I² = 51%								
Test for overall effect: Z = 0.55 (P = 0.59)								
**Calcitriol supplementation / High risk of bias**								
Grady 1991	0.64	1.9	50	1.51	2.4	48	23.8%	-0.87 [-1.73, -0.01]
**Subtotal (95% CI)**			**50**			**48**	**23.8%**	**-0.87 [-1.73, -0.01]**
Heterogeneity: Not applicable								
Test for overall effect: Z = 1.98 (P = 0.05)								
**Total (95% CI)**			**545**			**520**	**100.0%**	**-0.07 [-0.70, 0.55]**
Heterogeneity: Tau² = 0.24; Chi² = 8.12, df = 4 (P = 0.09); I² = 51%								
Test for overall effect: Z = 0.23 (P = 0.82)								
Test for subgroup differences: Chi² = 3.38, df = 1 (P = 0.07), I² = 70.4%								

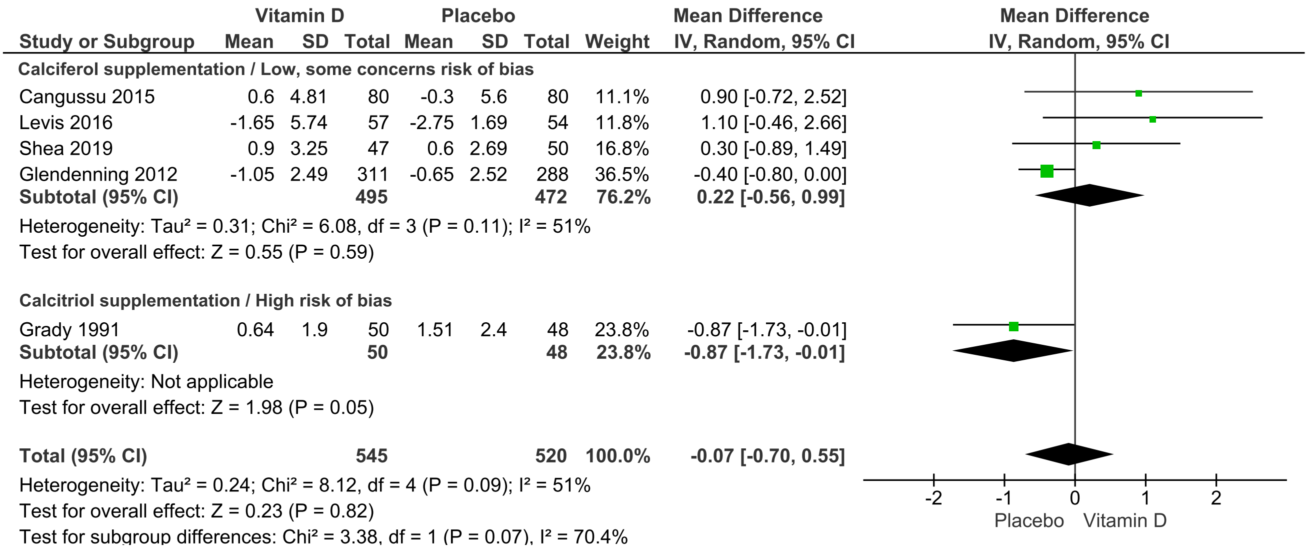

B

	Vitamin D			Placebo				Std. Mean Difference
**Study or Subgroup**	**Mean**	**SD**	**Total**	**Mean**	**SD**	**Total**	**Weight**	**IV, Random, 95% CI**
**Calciferol supplementation / Low, some concerns risk of bias**								
Pirotta 2015 (KET)	0.13	0.22	13	0.1	0.11	12	3.7%	0.16 [-0.62, 0.95]
Shea 2019 (HGS)	0.9	3.25	47	0.6	2.69	50	11.1%	0.10 [-0.30, 0.50]
Levis 2016 (HGS)	-1.65	5.74	57	-2.75	1.69	54	12.2%	0.26 [-0.12, 0.63]
Cangussu 2015 (HGS)	0.6	4.81	80	-0.3	5.6	80	15.3%	0.17 [-0.14, 0.48]
Aloia 2019 (HGS)	-14.3	319.25	130	-17.1	250.84	130	19.8%	0.01 [-0.23, 0.25]
Glendenning 2012 (HGS)	-1.05	2.49	311	-0.65	2.52	288	26.9%	-0.16 [-0.32, 0.00]
**Subtotal (95% CI)**			**638**			**615**	**89.0%**	**0.02 [-0.12, 0.17]**
Heterogeneity: Tau² = 0.01; Chi² = 7.10, df = 5 (P = 0.21); I² = 30%								
Test for overall effect: Z = 0.31 (P = 0.76)								
**Calcitriol supplementation / High risk of bias**								
Grady 1991 (HGS)	0.64	1.9	50	1.51	2.4	48	11.0%	-0.40 [-0.80, 0.00]
**Subtotal (95% CI)**			**50**			**48**	**11.0%**	**-0.40 [-0.80, 0.00]**
Heterogeneity: Not applicable								
Test for overall effect: Z = 1.96 (P = 0.05)								
**Total (95% CI)**			**688**			**663**	**100.0%**	**-0.01 [-0.17, 0.15]**
Heterogeneity: Tau² = 0.02; Chi² = 10.34, df = 6 (P = 0.11); I² = 42%								
Test for overall effect: Z = 0.13 (P = 0.90)								
Test for subgroup differences: Chi² = 3.78, df = 1 (P = 0.05), I² = 73.5%								

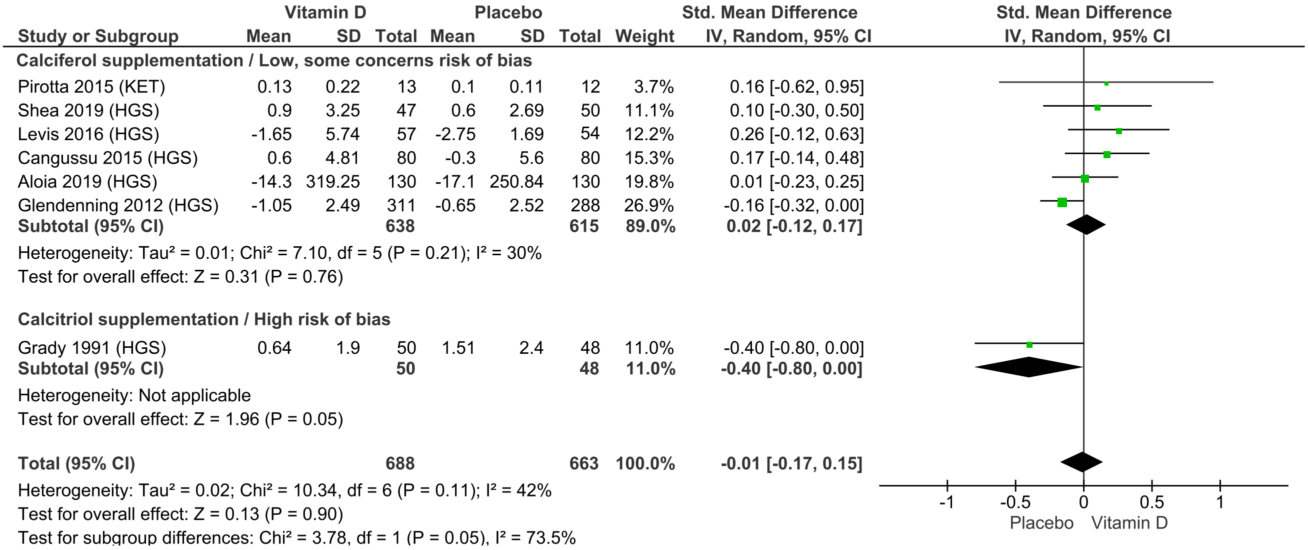

C

Study or Subgroup	Vitamin D Mean	Vitamin D SD	Vitamin D Total	Placebo Mean	Placebo SD	Placebo Total	Weight	Std. Mean Difference IV, Random, 95% CI
**Calciferol supplementation / Low, some concerns risk of bias**								
Pirotta 2015 [25(OH)D]	13.6	4.14	13	-0.48	2.39	13	7.4%	4.03 [2.62, 5.45]
Ceglia 2013 [25(OH)D]	14.56	6.44	9	1.68	5.52	12	8.5%	2.09 [0.97, 3.20]
Shea 2019 [25(OH)D]	13.2	4.97	47	-1	2.78	48	10.0%	3.51 [2.86, 4.16]
Glendenning 2012 [25(OH)D]	3.84	13.04	20	-2.52	9.61	20	10.1%	0.54 [-0.09, 1.18]
Hansen 2015 [25(OH)D]	21	8.78	79	-3	4.86	76	10.4%	3.35 [2.86, 3.84]
Levis 2016 [25(OH)D]	23.02	14.19	56	1.18	5.83	55	10.5%	1.99 [1.54, 2.45]
Lips 2010 [25(OH)D]	26.4	7	99	13.5	7.7	97	10.8%	1.75 [1.42, 2.08]
Cangussu 2015 [25(OH)D]	12.48	23.54	80	-3.14	9.92	80	10.8%	0.86 [0.54, 1.18]
Aloia 2019 [25(OH)D]	25.3	15.23	130	-1.8	9.9	130	10.8%	2.10 [1.80, 2.41]
**Subtotal (95% CI)**			**533**			**531**	**89.3%**	**2.17 [1.54, 2.81]**
Heterogeneity: Tau² = 0.83; Chi² = 125.46, df = 8 (P < 0.00001); I² = 94%								
Test for overall effect: Z = 6.69 (P < 0.00001)								
**Calcitriol supplementation / High risk of bias**								
Grady 1991 [1,25(OH)D]	9.9	46.19	50	2.7	25.43	48	10.7%	0.19 [-0.21, 0.59]
**Subtotal (95% CI)**			**50**			**48**	**10.7%**	**0.19 [-0.21, 0.59]**
Heterogeneity: Not applicable								
Test for overall effect: Z = 0.94 (P = 0.35)								
**Total (95% CI)**			**583**			**579**	**100.0%**	**1.97 [1.30, 2.64]**
Heterogeneity: Tau² = 1.06; Chi² = 186.19, df = 9 (P < 0.00001); I² = 95%								
Test for overall effect: Z = 5.75 (P < 0.00001)								
Test for subgroup differences: Chi² = 26.82, df = 1 (P < 0.00001), I² = 96.3%								

Std. Mean Difference
IV, Random, 95% CI

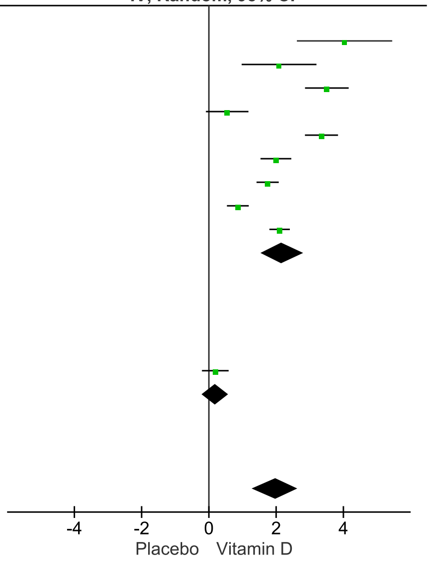

Supplement: Supplementary file 1 — Table S1. Search terms employed to screen different electronic databases for the literature search. [file JCSM-13-1642-s002.pdf]
