# Supplementary material for: Effect of vitamin D monotherapy on indices of sarcopenia in community‐dwelling older adults: a systematic review and meta‐analysis
Source: J Cachexia Sarcopenia Muscle. 2022 Mar 8;13(3):1642–52. doi: 10.1002/jcsm.12976 (PMC9178168; doi:10.1002/jcsm.12976)
Supplement: Supplementary file 2 — Figure S1. Quality assessment of the included studies according to the Cochrane risk‐of‐bias 2 tool. Figure S2. Effect of vitamin D supplementation on changes in serum vitamin D (25‐hydroxyvitamin D [25(OH)D] and 1,25‐dihydroxyvitamin D [1,25(OH)2D]) levels compared to placebo. Figure S3. Subgroup analysis of handgrip strength changes in response to vitamin D supplementation based on (A) sex, vitamin D treatment (B) duration and (C) dose and (D) geographic origin of study, compared to placebo. Figure S4. Sensitivity analysis based on the effect of lifestyle factors on changes in (A) handgrip strength (B) serum vitamin D (25‐hydroxyvitamin D [25(OH)D] and 1,25‐dihydroxyvitamin D [1,25(OH)2D]), in response to vitamin D supplementation compared to placebo. Figure S5. Sensitivity analysis based on the effect of type of vitamin D supplementation or risk of bias on changes in (A) handgrip strength (HGS), (B) general muscle strength (HGS and knee extension test at 180 degrees (KET)) and (C) serum vitamin D (25‐hydroxyvitamin D [25(OH)D] and 1,25‐dihydroxyvitamin D [1,25(OH)2D]) levels, in response to vitamin D supplementation compared to placebo. [file JCSM-13-1642-s001.pdf]

Supplementary Tables

Table S1. Search terms employed to screen different electronic databases for the literature search.

Database	Search terms
PubMed	(“vitamin D” OR “vitamin D2” OR “vitamin D3” OR “1-alpha hydroxyvitamin D3” OR “1-alpha hydroxycalciferol” OR “1,25-dihydroxyvitamin D3” OR “1,25 dihydroxycholecalciferol” OR “25- hydroxycholecalciferol” OR “25 hydroxyvitamin D” OR “calcitriol” OR “ergocalciferol” OR “cholecalciferol” OR “calcifediol” OR “calcidiol” OR “calciferol”) AND (“Sarcopenia” OR “handgrip strength” OR “gait speed” OR “muscle” OR “muscle mass” OR “muscle loss” OR “muscle strength” OR “frailty” OR “Short Physical Performance Battery” OR “skeletal muscle mass” OR “appendicular muscle mass” OR “appendicular lean mass” OR “chair stand test” OR “sit-stand-test” OR “knee extension” OR “knee flexion” OR “quadriceps strength” OR “lower limb strength” OR “stair climb”)
Cochrane Library	(“vitamin D” OR “1-alpha hydroxyvitamin D3” OR “1-alpha hydroxycalciferol” OR “1,25-dihydroxyvitamin D3” OR “1,25 dihydroxycholecalciferol” OR “25- hydroxycholecalciferol” OR “25 hydroxyvitamin D” OR “calcitriol” OR “ergocalciferol” OR “cholecalciferol” OR “calcifediol” OR “calcidiol” OR “calciferol”) AND (“Sarcopenia” OR “handgrip strength” OR “gait speed” OR “muscle mass” OR “muscle strength” OR “frailty” OR “Short Physical Performance Battery” OR “skeletal muscle mass” OR “appendicular lean mass” OR “chair stand test” OR “sit-stand-test” OR “knee extension” OR “knee flexion” OR “quadriceps strength” OR “lower limb strength” OR “stair climb”)
Web of Science	TS=(“vitamin D” OR “1-alpha hydroxyvitamin D3” OR “1-alpha hydroxycalciferol” OR “1,25-dihydroxyvitamin D3” OR “1,25 dihydroxycholecalciferol” OR “25- hydroxycholecalciferol” OR “25 hydroxyvitamin D” OR “calcitriol” OR “ergocalciferol” OR “cholecalciferol” OR “calcifediol” OR “calcidiol” OR “calciferol”) AND (“Sarcopenia” OR “handgrip strength” OR “gait speed” OR “muscle mass” OR “muscle strength” OR “frailty” OR “Short Physical Performance Battery” OR “skeletal muscle mass” OR “appendicular lean mass” OR “chair stand test” OR “sit-stand-test” OR “knee extension” OR “knee flexion” OR “quadriceps strength” OR “lower limb strength” OR “stair climb”)
Scopus	TITLE-ABS-KEY (“vitamin D" OR “calcitriol" OR “ergocalciferol" OR “cholecalciferol" OR “calcifediol" OR “calcidiol" OR “calciferol”) AND (“handgrip strength" OR” gait speed" OR” SPPB" OR “appendicular lean mass" OR “chair stand test" OR” knee extension" OR “knee flexion" OR “quadriceps strength”)
